# Hypothesis: Mechanisms That Prevent Recovery in Prolonged ICU Patients Also Underlie Myalgic Encephalomyelitis/Chronic Fatigue Syndrome (ME/CFS)

**DOI:** 10.3389/fmed.2021.628029

**Published:** 2021-01-28

**Authors:** Dominic Stanculescu, Lars Larsson, Jonas Bergquist

**Affiliations:** ^1^Independent Researcher, Sint Martens Latem, Belgium; ^2^Basic and Clinical Muscle Biology, Department of Physiology and Pharmacology, Karolinska Institute, Solna, Sweden; ^3^Analytical Chemistry and Neurochemistry, Department of Chemistry – Biomedical Center, Uppsala University, Uppsala, Sweden; ^4^The Myalgic Encephalomyelitis/Chronic Fatigue Syndrome (ME/CFS) Collaborative Research Centre at Uppsala University, Uppsala, Sweden

**Keywords:** myalgic encephalomyelitis, critical illness, non-thyroidal illness syndrome, low t-3 syndrome, pituitary, cytokines, oxidative and nitrosative stress, post-intensive care syndrome

## Abstract

Here the hypothesis is advanced that maladaptive mechanisms that prevent recovery in some intensive care unit (ICU) patients may also underlie Myalgic encephalomyelitis/chronic fatigue syndrome (ME/CFS). Specifically, these mechanisms are: (a) suppression of the pituitary gland's *pulsatile* secretion of tropic hormones, and (b) a “vicious circle” between inflammation, oxidative and nitrosative stress (O&NS), and low thyroid hormone *function*. This hypothesis should be investigated through collaborative research projects.

## Introduction

Critical illness refers to the physiological response to virtually any severe injury or infection, such as sepsis, liver disease, HIV infection, head injury, pancreatitis, burns, cardiac surgery, etc. (1). Researchers make a distinction between the *acute* phase of critical illness—in the first hours or days following severe trauma or infection; and the *chronic* or *prolonged* phase—in the case of patients that survive the *acute* phase but for unknown reasons do not start recovering and continue to require intensive care (i.e., “chronic ICU patients”). Independent of the nature of the critical illness, the *acute* phase is associated with an excessive response of pro-inflammatory cytokines (2) and is characterized by a uniform dysregulation of the endocrine axes (3). In *prolonged* critical illness, this dysregulation is maintained even once the initial inflammatory surge has settled (4). Regardless of the initial injury or infection, patients that suffer from *prolonged* critical illness experience profound muscular weakness, cognitive impairment, loss of lean body mass, pain, increased vulnerability to infection, skin breakdown, etc. (1, 5, 6). Whereas, the *acute* phase is considered to be an *adaptive* response to the severe stress of injury or infection (shifting energy and resources to essential organs and repair), the physiological mechanisms in the *prolonged* phase are now increasingly considered to be *maladaptive* responses to the stress of severe injury or infection, hindering recovery (7–10). Some have also suggested that the non-recovery from endocrine disturbances could explain the development of “post-intensive care syndrome” (PICS) (11); i.e., “the cognitive, psychiatric and/or physical disability after treatment in ICUs” (12, 13).

Myalgic encephalomyelitis/chronic fatigue syndrome (ME/CFS) is a debilitating, multi-system disease of unclear etiology (14, 15). The most common peri-onset events reported by patients are infection-related episodes (64%), stressful incidents (39%), and exposure to environmental toxins (20%) (16). “Impaired function, post-exertional malaise (an exacerbation of some or all of an individual's ME/CFS symptoms after physical or cognitive exertion, or orthostatic stress that leads to a reduction in functional ability), and unrefreshing sleep” are considered to be core symptoms (14). The severity of the symptoms varies: “very severely affected patients experience profound weakness, almost constant pain, severe limitations to physical and mental activity, sensory hypersensitivity (light, touch, sound, smell, and certain foods), and hypersensitivity to medications” (17). We have listed a few hall mark symptoms that are often found in critically ill patients in chronic intensive care (ICU) patients and ME/CFS patients (Table 1).

**Table 1:** Comparison of the typical clinical picture of ICU patients and patients with ME/CFS.

**Type**	**Prolonged critical illness (5)**	**ME/CFS (18, 19)**
Neuromuscular/Neurological	**“Profound weakness** attributed to myopathy, neuropathy, and alterations of body composition including loss of lean body mass, increased adiposity, and anasarca” **“Ventilator dependence”**	• Extreme fatigue or lack of energy • Persistent exhaustion • Post-exertional malaise (symptoms worsen after exertion) • Ataxia and muscle weakness • Pain (muscle and joint pains) • Orthostatic intolerance
Endocrine/Autonomic	**“Distinctive neuroendocrine changes** including loss of pulsatile secretion of anterior pituitary hormones, contributing to low target organ hormone levels and impaired anabolism”	• Temperature instability • Weight loss or gain • Sleep dysfunction & unrefreshing sleep • Postural Orthostatic Tachycardia Syndrome (POTS) • Light-headedness • Change in appetite • Nausea & irritable bowel syndrome
Immunological	**“Increased vulnerability to infection**, often with multi-resistant microbial organisms” **“Skin breakdown** associated with nutritional deficiencies, edema, incontinence, and *prolonged* immobility”	• Infection-immune like symptoms • Recurrent flu-like symptoms • Sweating/Fever • New sensitivities to food, medication, chemicals • Sore throat • Tender lymph nodes
Cognitive	**“Brain dysfunction** manifesting as coma or delirium that is protracted or permanent”	• Cognitive impairment • Brain Fog • Confusion and Disorientation • Difficulty concentrating • Short-term memory issues • Hypersensitivity to noise and light
Emotional	**“Distress** from symptoms including pain, thirst, dyspnea, depression, and anxiety”	• Emotional instability, anxiety, and depression

Here the hypothesis is advanced that maladaptive mechanisms that prevent recovery in some ICU patients also underlie ME/CFS. Specifically, these mechanisms are: (a) suppression of the pituitary gland's *pulsatile* secretion of tropic hormones, and (b) a “vicious circle” between inflammation, oxidative and nitrosative stress (O&NS), and low thyroid hormone *function*. These mechanisms characterize *prolonged* critical illness regardless of the nature of the initial severe injury or infection (3, 8–10); similarly, we propose that these mechanisms could underlie the perpetuation of illness in ME/CFS regardless of the nature of the peri-onset event (i.e., infection, stressful incident, exposure to environmental toxins, or other). We provide an overview of these mechanisms in ICU patients and discuss their relevance for understanding ME/CFS. We also bring findings from fibromyalgia into the discussion here because ME/CFS and fibromyalgia are often jointly considered in the literature (20, 21); fibromyalgia is similarly a syndrome that is medically unexplained, often comorbid with ME/CFS, and “shares the core symptoms of fatigue, sleep problems and cognitive difficulties” (22). Additional research projects are required to investigate the validity of this hypothesis building on the findings from critical illness and ME/CFS summarized here.

This hypothesis may be particularly relevant in light of the current COVID-19 pandemic. Many COVID-19 patients continue to experience a variety of debilitating symptoms despite successfully defeating the virus—termed “post COVID-19 syndrome” or “long COVID-19”—that resemble ME/CFS (23–26).

## Suppression of *Pulsatile* Pituitary Secretions

Endocrine patterns observed during the initial *acute* phase of critical illness (in the first few hours or days) differ markedly from those observed during *prolonged* critical illness (after a few days) (27, 28). Indeed, the *acute* phase is characterized by *increased* release of pituitary hormones; the *prolonged* phase is characterized by *suppression* of the release of pituitary hormones. Simultaneously, hormone half-life and hormone up-take by the peripheral tissues differ markedly between these two phases (4, 29). This *biphasic* pattern of the endocrine system during critical illness, however, is not readily observable in single or average measurements of circulating tropic and non-tropic hormone concentrations—which are a function of both hormone release and elimination from the blood stream. This pattern was thus only discovered in the early 1990s with measurements of the *frequency* and *amplitude* of pituitary secretions (i.e., *pulsatility*) performed as often as every 10 min over 24 h on ICU patients (29). The *pulsatility* of tropic hormone secretion is part of the signaling to the peripheral glands and thus considered a determining factor of hormone *function* (i.e., impact on target glands or tissues), in addition to overall volume of hormone release (30, 31). The finding that *pulsatile* pituitary secretions are suppressed during *prolonged* critical illness was critical in understanding the physiology of the syndrome and the curious failure of patients to recover (32). We describe the biphasic endocrine patterns during *acute* and *prolonged* critical illness for each of the main endocrine axes in further detail below, as well as the implications for the autonomic nervous system, metabolism and the immune system. We also provide evidence suggesting that the endocrine patterns observed in *prolonged* critical illness also underlie ME/CFS.

### The Adreno-Cortical Axis (HPA Axis)

The adreno-cortical axis—also called hypothalamic-pituitary-adrenal (HPA) axis—is the body's primary stress management system. The HPA axis responds to physical and mental challenges in part by controlling the body's glucocorticoids levels, notably cortisol (33). Cortisol in turn modulates inflammation response, cardiovascular function and glucose metabolism (34). An inability to deal with stress, proneness to exaggerated immune responses and weight loss are associated with hypocortolism or poor HPA axis function (35–38). The HPA axis also regulates mineralocorticoids that, in turn, regulate water and electrolyte balance (i.e., blood pressure). Low blood pressure and dizziness upon standing up are associated with a compromised HPA axis (35). Finally, the HPA axis (in addition to the gonadotropic axis not covered here) also contributes to the production of androgens, notably DHEA and testosterone, which are steroids that impact muscle mass, fat storage, pain, brain function and many other physiological traits. Low androgens are associated with muscle fatigue, joint pain, and noise intolerance (39–42).

In normal conditions, the adrenal gland secretes cortisol during the day in pulses, with the highest amounts in the early morning hours and lower amounts at night. The hypothalamus signals to the pituitary with corticotrophin-releasing hormone (CRH), and to a lesser extent, arginine vasopressin (AVP), to produce adrenocorticotropic hormone (ACTH). This is in turn signals the adrenals to release cortisol and other hormones. Most cortisol circulating in the blood is bound to carrier molecules (29, 43). Production of cortisol is regulated by an inhibitory feedback loop. When free circulating cortisol attaches to glucocorticoid receptors on the hypothalamus and pituitary, these glands reduce production of CRH and AVP, and ACTH, respectively. The number and affinity of glucocorticoid receptors is thus considered one of the most important determining factors in the regulation of the HPA axis (43)

#### In Critical Illness

During the *acute* phase of critical illness, plasma cortisol concentrations rise rapidly. Increased cortisol availability is considered a vital response that allows for fluid retention, increased cardiac output and blood pressure, and induces an appropriate immune response while protecting against excessive inflammation (29, 44, 45). Until recently believed to be the result of increased cortisol production by the adrenals, it is now known that high cortisol availability during this phase of critical illness is in fact largely driven by two peripheral mechanisms: a decrease in the abundance and affinity of the cortisol carrier molecules in circulation, and a slowing of cortisol breakdown in the liver and kidney (29, 34, 44, 46, 47). Via inhibitory feedback loops, these higher cortisol concentrations suppress the HPA axis at the central level: the secretions of CRH and AVP by the hypothalamus and of ACTH by the pituitary fall, leading to an eventual drop in plasma cortisol levels (48).

Whereas, in critically ill patients that begin to recover, the HPA axis essentially normalizes within 28 days of illness, in cases of *prolonged* critical illness ACTH levels (surprisingly) continue to be depressed despite dropping cortisol levels (49, 50). Why and how this central suppression of ACTH is maintained is not clear and continues to be debated. Pro-inflammatory cytokines and O&NS likely play a leading role. Cytokines can mediate tissue-specific changes in the abundance and affinity of glucocorticoid receptors—which are major factors determining the activity of the HPA axis (2, 44). Specifically, the cytokine IL-1β is known to modulate CRH release by the hypothalamus; TNF-α is known to impair ACTH release by the pituitary; and TNF-α is also known to impair cortisol production by the adrenal glands (2).

**Figure 1:**
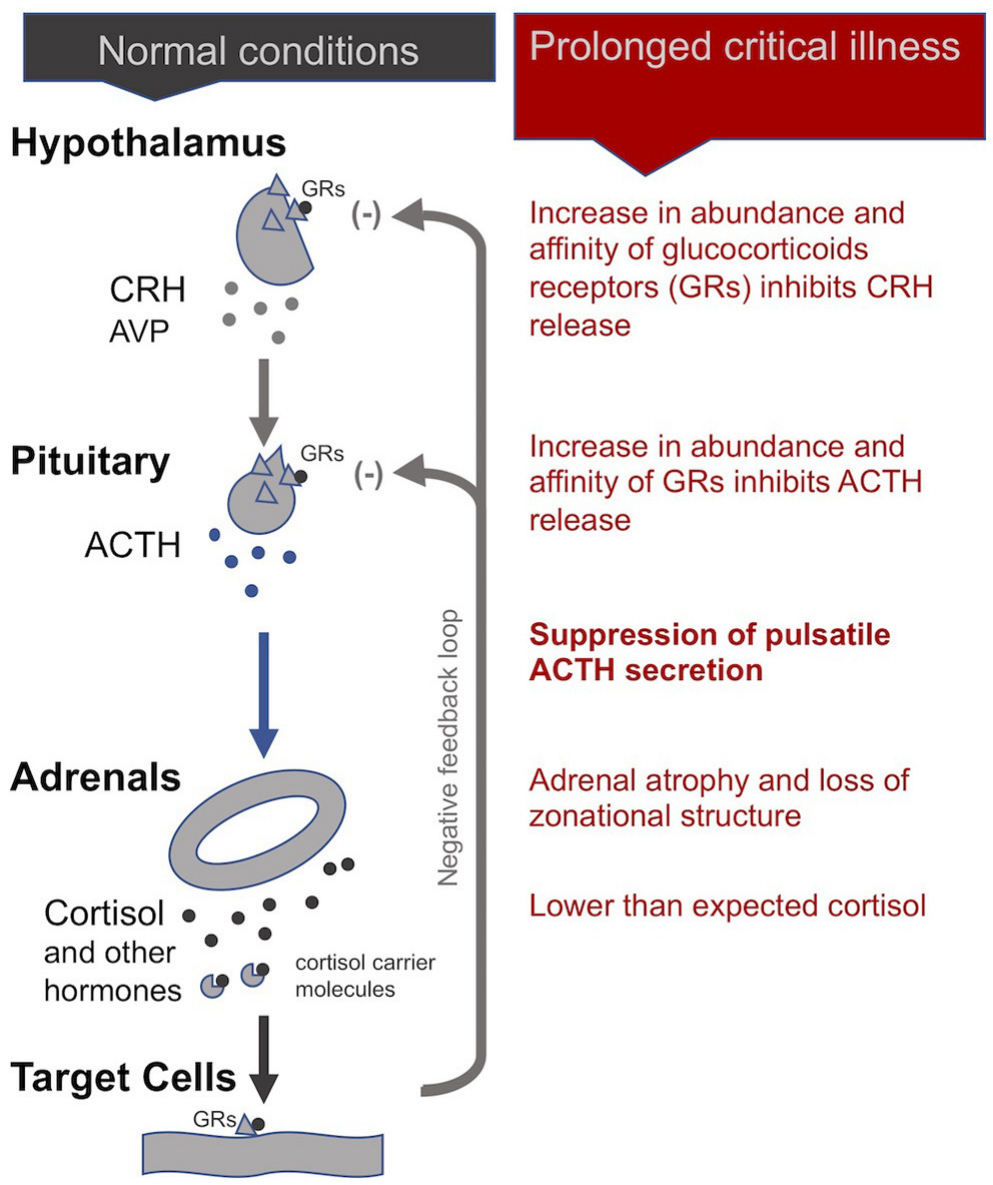
The adreno-cortical axis (HPA axis) during normal conditions and *prolonged* critical illness.

Without sufficient *pulsatile* stimulation by the tropic hormone ACTH, adrenal glands begin to atrophy and lose zonational structure. This is evidenced in the post-mortem dissection of patients that had been critically ill for a few weeks, but not in the patients that quickly died from their illness or trauma (34, 51). The weakening of adrenal glands not only compromises patients' ability to cope with external stressors but also permits excessive inflammatory responses. In sum, the initial beneficial increase in cortisol availability induced by peripheral mechanisms during the *acute* phase of critical illness leads to a suppression of the HPA axis at the central-level from which a subset of patients appears unable to escape (Figure 1).

#### In ME/CFS

Dysfunction of the HPA axis has been documented extensively in ME/CFS patients since the early 1980s (52–63). Researchers have observed decreased baseline cortisol levels, blunted HPA axis responses to physical and psychological stressors, reduced HPA axis responsivity to provocation tests (such as CRH and ACTH administration), and a heightened inhibitory feedback loop (consistent with a higher abundance and affinity of glucocorticoid receptors at the level of the pituitary and hypothalamus). Strikingly, the magnitude of HPA axis dysfunction becomes more pronounced with illness duration and is associated with symptom severity (43, 64). Very few have studied *pulsatility* of ACTH release: one study of 36 study-pairs found no statistically significant differences in ACTH *pulsatility* between ME/CFS and matched controls (65), while another found a differential pattern of ACTH release over 24-h periods (66). Variations in the study-participants' severity of illness—and methods used to control for these—may explain these apparently contradictory findings. Several studies have found the morning peak of ACTH is missing or weak in ME/CFS patients (43). A recent study assessing secretory events of cortisol found that CFS/ME patients have the same number of secretory events but secrete lower quantities in early morning hours (67). Significantly, a group of ME/CFS patients were found to have 50% smaller adrenals than controls (68), resembling adrenal atrophy in *prolonged* critical illness.

ME/CFS researchers have also proposed models to explain the persistence of a suppressed HPA axis (33, 69, 70). Essentially, a short stress (i.e., a burst of cortisol) will produce a small perturbation in the glucocorticoid receptor concentration on the central glands that quickly returns to normal levels. However, long, repeated stress—from which the system doesn't have time to recover—leads to a persistent high glucocorticoid receptor concentration, forcing the HPA axis to an alternate steady state. More recent models of the HPA axis have also included non-genomic feedback-controls (71), the endogenous effects of circadian rhythm (72), and interactions with the gonadotropic axis and the immune system (73, 74) to explain how HPA axis suppression is maintained even after the initial stress is gone.

HPA axis dysfunction is also present in the majority of fibromyalgia patients (75–77). Various mechanisms have been suggested, including depressed secretion of CRH by the hypothalamus, a deficiency of CRH receptors on the pituitary, and adrenal atrophy due to chronic under-stimulation by reduced ACTH levels (78).

Moreover, the dysfunction of the HPA axis in ME/CFS and fibromyalgia has also been associated with pro-inflammatory cytokines and O&NS (43, 55, 79, 80). A recent paper considering the bidirectional relationship between the function of the HPA axis and inflammation finds that immune-inflammatory and O&NS pathways *induce* HPA axis dysfunction in ME/CFS (81); the direction of causality is analogous to inflammatory pathways inducing endocrine dysfunctions in critical illness. Others have similarly theorized that local inflammation in the hypothalamus leads to a disturbed HPA axis in ME/CFS (82).

In sum, the HPA axis dysfunctions in ME/CFS are not unlike the dysfunctions in *prolonged* critical illness. However, to our knowledge a comprehensive study of the pituitary *pulsatile* secretions of ACTH in ME/CFS patients—which proved revelatory in understanding *prolonged* critical illness—does not yet exist. The relationship between the pituitary's *pulsatile* ACTH secretions, severity of illness, the integrity and function of adrenal glands and resulting physiological alterations in ME/CFS thus remains largely unexplored.

### The Somatotropic Axis (HPS Axis)

The somatotropic axis—also called hypothalamic-pituitary-somatotropic (HPS) axis—plays important roles in growth and development of children, but also contributes to a variety of physiological pathways in adults, including balancing *catabolic* (i.e., the break-down of molecules and tissues) and *anabolic* activities (i.e., the building of molecules and tissue) (4). An HPS axis dysfunction is known to cause loss of muscle and bone mass, induces weakness (29), and impacts gut mucosa integrity as well as glucose and fat metabolism (83). Low energy, exhaustion, mental fatigue, weak muscle strength as well as poor recovery after physical activity are associated with an inhibited HPS axis function (42, 84, 85).

Uniquely, in the case of the HPS axis, the hypothalamus sends both stimulating (+) and inhibiting (-) signals to the pituitary for the production of growth hormone (GH): these are, respectively, the GH-releasing hormone (GHRH) and the GH-inhibiting hormone (GHIH, also called somatostatin) (4). In addition, ghrelin, mostly produced by the gut, also stimulates GH production by the pituitary. In normal conditions, GH is released by the pituitary in a *pulsatile* fashion under the control of these three signals, with peaks of GH levels alternating with virtually undetectable valleys in 3- to 5-h intervals over the course of the day (29). GH in turn has direct effects on some tissues and also stimulates the production of insulin-like growth hormone-1 (IGF-1), mostly by the liver. Nearly all of the IGF-1 hormones in the plasma are bound to IGF-binding proteins (IGFBP). IGF-1 and GH exert inhibitory feedback on the hypothalamus and the pituitary to maintain homeostasis. The half-life of GH is only 10 to 20 min, whereas the half-life of IGF-1 is more than 12 h. Thus, IGF-1 plasma concentrations are regularly used as proxies for GH secretion in clinical settings. This, however, overlooks the function of the *pulsatile* secretion of GH on the balance of anabolic and catabolic activities in the body (4).

#### In Critical Illness

In the *acute* phase of critical illness, the pituitary produces more GH: higher peaks, lower valleys and increased pulse frequencies (86). The rapid onset of two main peripheral mechanisms explain this finding: First, under the influence of cytokines, the liver expresses fewer GH receptors (i.e., becomes resistant to GH) and thus produces less IGF-1. Second, alterations in IGF binding proteins results in IGF-1 being cleared out faster from the system (i.e., IGF-1 has a shorter half-life) (87). The lower IGF-1 concentrations resulting from these two peripheral mechanisms will—via the feedback loop inherent to the axis—spur more GH production (29). The resulting increase in *catabolic* activity during the *acute* phase of critical illness serves to mobilize amino acids derived from the breakdown of peripheral tissues, such as skeletal muscle and bone, for use by the central organs (4).

However, if a critically ill patient fails to recover within a few days, GH secretion becomes *erratic* and almost non-pulsatile. Experiments have demonstrated that this is largely due to a lack of stimulation of the hypothalamus and pituitary by the hormone ghrelin. There is also evidence for changes in the relative amounts of GHIH and GHRH signals from the hypothalamus (4). As for the peripheral hormone, IGF-1, its levels are low or normal in *prolonged* critical illness. The liver's resistance to GH (which previously suppressed IGF-1 production during the *acute* phase of critical illness) does not persist during *prolonged* critical illness (29, 87). However, without a concomitant pulsatile release of GH, the *anabolic* function of IGF-1 becomes inhibited (4).

**Figure 2:**
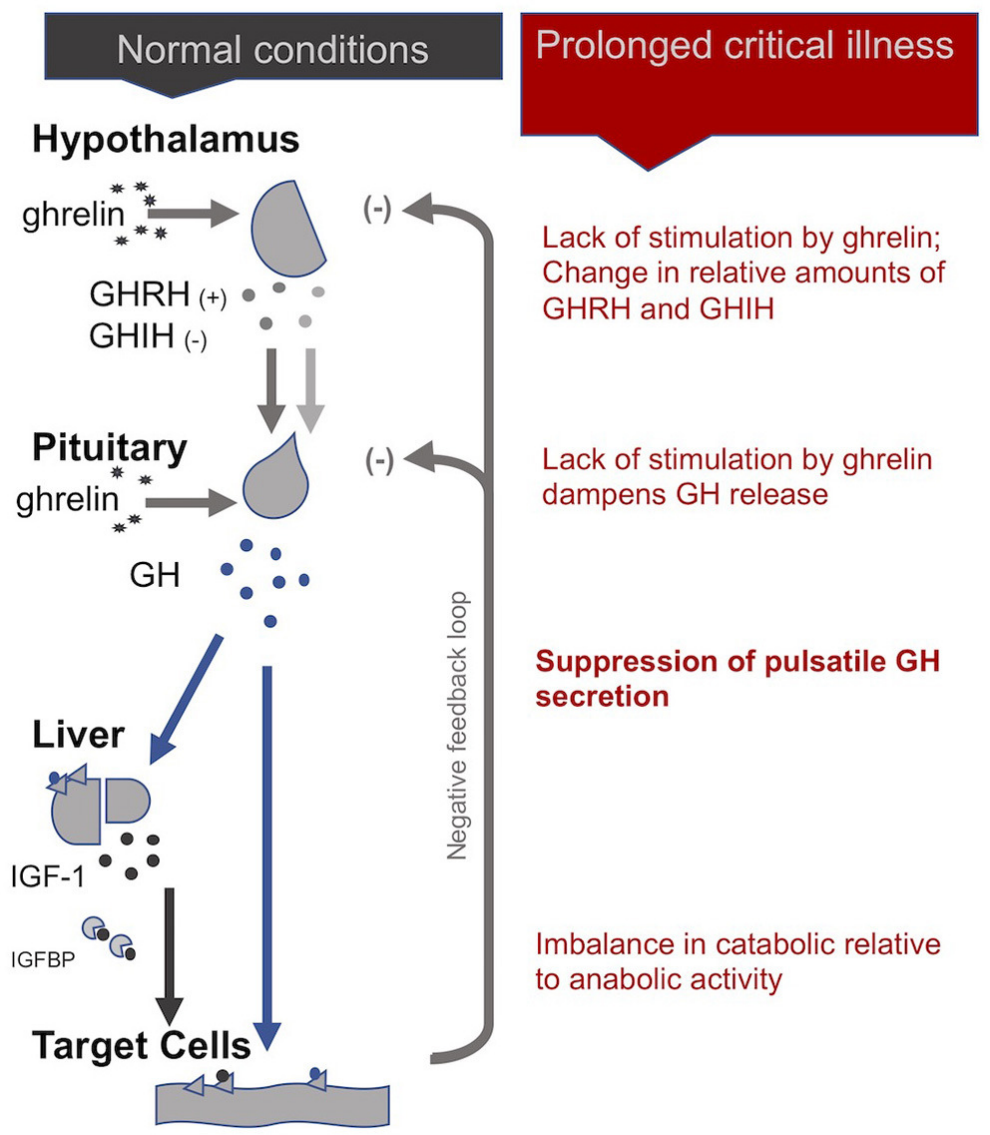
The somatotropic axis (HPS axis) during normal conditions and *prolonged* critical illness.

In sum, although the increase in catabolic activity during the *acute* phase of critical illness may initially be beneficial because it serves to mobilize amino acids, the perpetuation of the imbalance in catabolic vs. anabolic activity (due in part to the loss of the pulsatile function of GH) during *prolonged* critical illness may be considered maladaptive (Figure 2). The imbalance in catabolic relative to anabolic activity in *prolonged* critical illness leads to protein break-down in skeletal muscle, liver, kidney and heart, reducing their cell mass and leading to impaired function (7). These processes are ultimately reflected in muscle and bone wasting typically present in *prolonged* critical illness (88, 89).

#### In ME/CFS

GH regulation in ME/CFS has been studied since the 1990s. The findings are mixed, but almost none addresses the question of the *pulsatility* of GH release. Some described low nocturnal GH secretion (90, 91), while others have found normal levels of 24-h urinary GH excretion (92). Some have found reduced response to induced hypoglycemia (90, 91), while others describe normal GH responses to stimulation (93). One study describes unaffected diurnal patterns of GH release in ME/CFS, but it focused on assessing basal levels rather than the nature of secretory patterns (i.e., pulsatile vs. erratic) and may not have accounted for variations in the severity of illness of patients (66). In terms of IGF-1, there are no consistent differences between ME/CFS patients and controls (93, 94), which is consistent with findings from *prolonged* critical illness.

Studies in fibromyalgia show relative GH deficiency (76, 78, 95–99) and low or low-normal IGF-1 levels (95, 96, 100). Interestingly, some studies showed that fibromyalgia patients “failed to exhibit a GH response to exercise” (97, 101), consistent with a loss in *pulsatility* of GH release.

In sum, endocrine observations in ME/CFS are not unlike HPS axis dysfunctions found in *prolonged* critical illness. To our knowledge the pituitary *pulsatile* secretions of GH in ME/CFS patients has not been comprehensively studied. The relationship between the pituitary's *pulsatile* GH secretions, severity of illness and the balance between catabolic and anabolic activities in ME/CFS thus remains largely undiscovered.

### The Thyrotropic Axis (HPT Axis)

The thyrotropic axis—also called hypothalamic-pituitary-thyroid (HPT) axis—regulates the basal rate of our metabolism. Dysfunctions of the HPT axis are associated with tiredness, stiffness, constipation, dry skin and weight gain, among a myriad of other hypothyroid-like symptoms (35, 42).

In normal conditions, an inhibitory feedback loop works to maintain stable circulating thyroid hormone concentrations according to a daily rhythm (102). When unbound circulating thyroid hormone concentrations dip below a certain threshold, the hypothalamus produces thyrotropin-releasing hormones (TRH) in order to signal the pituitary to produce thyroid stimulating hormone (TSH), which in turn signals the thyroid gland to produce more thyroid hormones.

#### In Critical Illness

Dysfunctions of the HPT axis during critical illness have been studied extensively. Starting in the early 1970s, clinicians working in ICUs observed that patients with a wide range of critical conditions had low plasma concentrations of the *active* form of thyroid hormones (T3) relative to plasma concentrations of *inactivated* thyroid hormones reverse T3 (rT3) (103–105). They gave this condition the name “non-thyroidal illness syndrome” (NTIS), also called “euthyroid sick syndrome” or “low T3 syndrome.” While NTIS was initially considered to be beneficial in critical illness—i.e., a state of “protective” down-regulation of metabolism during times of duress (106) —it is increasingly seen as maladaptive and hampering the recovery of patients in the case of *prolonged* critical illness (9, 10, 29, 103, 104, 107, 108).

During *acute* and early stages of critical illness, peripheral mechanisms involving cytokines (notably IL-1β, IL-6, TNF-α) lead to the quick depression of thyroid hormone activity (104, 105, 109–111) to help conserve energy resources (48, 104). The mechanisms include the alterations in the amount and affinity of thyroid hormone binding globulines in the blood (112–114); modifications in the expression of the transporters that carry the thyroid hormone into the cells (115, 116); the down- and up-regulation of deiodinase enzymes that convert the thyroid hormone into active and inactive forms, respectively (113, 117); and the variation in the quantity and isoforms of cellular thyroid hormone receptors present (notably in the liver, adipose tissue and muscle) (118–120). An alteration in any of these steps—which determine thyroid hormone *function*—can lead to large time- and tissue-specific adjustments in cellular metabolism (121, 122)—even without, or with only minor, changes in the blood concentrations of thyroid hormones (121, 123, 124).

During *prolonged* critical illness these peripheral mechanisms are supplemented by central mechanisms that also depress thyroid hormone *function* (125, 126). Cytokines (notably IL-12 and IL-18), in association with other signaling factors (including leptin, glucocorticoids, etc.), are believed to up-regulate the deiodinase enzymes D1 and D2 in the hypothalamus resulting in higher local levels of T3 that inhibit TRH release irrespective of circulating thyroid hormone concentrations (10, 127, 128). Moreover, cytokines (notably IL-1b and TNF-α) also suppress the release of TSH by the pituitary (129, 130). Finally, by reducing iodine uptake and thyroid hormone excretion, cytokines (notably IL-1) also impact the activity of the thyroid gland itself (103, 113). Together, these mechanisms can alter the inhibitory feedback mechanisms of the HPT axis (i.e., its “set-point”) during *prolonged* critical illness. Single measurements of circulating TSH, however, are ineffective in revealing such alterations in the set-point of the HPT axis.

**Figure 3:**
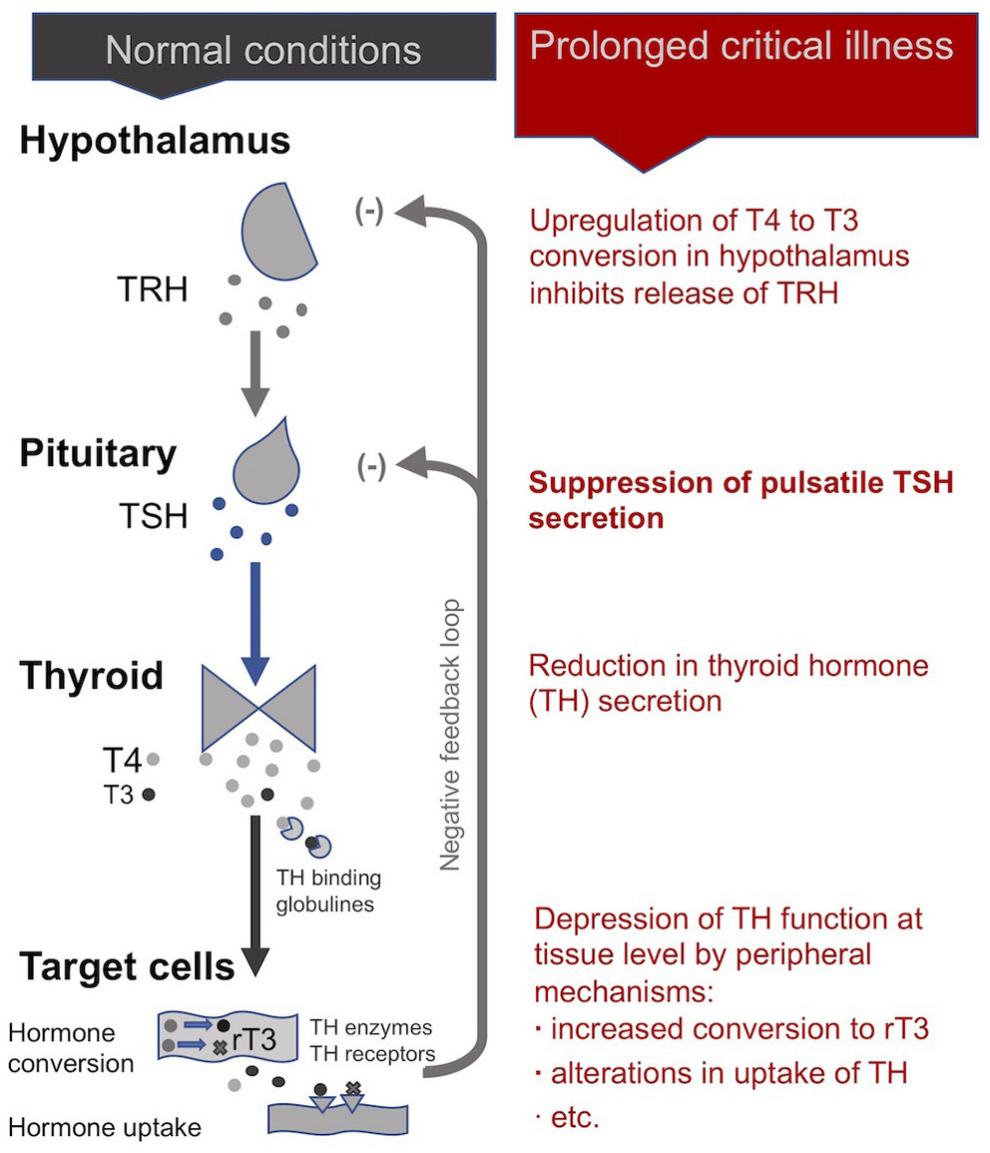
The thyrotropic axis (HPT axis) during normal conditions and *prolonged* critical illness.

In sum, an initial beneficial alteration of thyroid hormone activity in the periphery during *acute* critical illness is followed by a cytokine-mediated central suppression of the HPT axis resulting in a virtual complete loss of *pulsatile* TSH secretion (29). Peripheral mechanisms (notably variations in the conversion and transport of thyroid hormones) may further modulate thyroid hormone *function* in time- and tissue-specific ways resulting in complex physiological alterations in these patients (Figure 3) —not readily observable in blood concentrations of thyroid hormones. How these alterations of the HPT axis persist as well as their broader implications on metabolism and the immune system are further described below (see section A “Vicious Circle” Perpetuating Illness).

#### In ME/CFS

Dysfunctions of the HPT axis have long been suspected to play a role in ME/CFS (77, 131–134) and fibromyalgia (135–140). A recent study showed that ME/CFS patients had similar TSH levels as controls, but lower Free T3, Total T4, and Total T3, which the authors suggest resembles NTIS (141)—the typical feature of critically ill patients in ICUs described above.

In sum, alterations of the HPT axis in ME/CFS resemble dysfunctions found in *prolonged* critical illness. However, there does not to our knowledge exist a thorough study of the *pulsatility* of pituitary TSH secretion events in ME/CFS patients, nor a study of the tissue-specific alterations in thyroid hormone *function*—which proved revelatory in understanding *prolonged* critical illness. The relationship between the TSH axis dysfunctions, severity of illness, hypometabolic state and organ/tissue specific symptoms in ME/CFS thus remains largely unexplored.

### Intermediate Conclusions

The endocrine axes control many of the most fundamental physiological processes; their suppression is associated with a myriad of symptoms (see Table 2). Essentially, the suppression of *pulsatile* pituitary secretions of ACTH, GH, and TSH are central to *prolonged* critical illness. Inflammatory pathways play a role in inducing and maintaining this suppression irrespective of the nature of the original illness or trauma (see Table 3). The resulting endocrine patterns may be considered maladaptive and have wide ranging implications, including dysfunction of the balance between anabolic and catabolic processes, metabolism, and the regulation of the immune system. The physiological parallels between ME/CFS and *prolonged* critical illness would suggest that the suppression of *pulsatile* pituitary secretions of these tropic hormones might also underlie ME/CFS, and that the severity of ME/CFS might be a function of the strength of the mechanism; this however remains largely unstudied. In the next section we provide an overview of a model from critical illness that explains the perpetuation of these endocrine dysfunctions and we describe the relevance of the model for understanding ME/CFS.

**Table 2:** Summary of endocrine axes and function of the main hormones in adults.

**Name of Axis**	**Peripheral endocrine glands**	**Main hormones**	**Function**	**Symptoms of suppressed function**
Adreno-cortical axis: “HPA Axis”	Adrenal glands	Glucocorticoids, notably cortisol	Stress response via changes in glucose metabolism. Regulation of immune system.	Inability to deal with stress. Proneness to exaggerated immune responses. Weight loss.
		Mineralocorticoids, notably aldosterone	Regulate water and electrolyte balance (blood pressure).	Low blood pressure. Dizzy on standing up.
		Androgens, notably DHEA (can also be derived from gonadotropic axis).	Function as steroids on muscle mass, fat storage, brain function, etc.	Muscle fatigue. Noise intolerance.
Somatotropic axis: “HPS Axis”	Liver (mostly)	Growth hormone (GH) (produced by the pituitary) and IGF-1 (by the liver).	Regulation of insulin sensitivity, protein building (anabolic activity) and gut mucosal function.	Low energy and weak muscle strength. Poor recovery after physical activity. Exhaustion. Anxiety.
Thyrotropic axis: “HPT Axis”	Thyroid gland	Thyroid hormones: T4, T3, T2, T1, and reverse T3 (rT3).	Regulate baseline level of metabolism.	“Hypothyroid-like” symptoms: tiredness, stiffness, constipation, dry skin, etc. Weight gain.

**Table 3:** Summary of endocrine dysfunctions and mechanisms in critical illness and ME/CFS.

**Name of Axis**	**Dysfunctions** Prolonged critical illness and ME/CFS	**Mechanisms** Prolonged critical illness
HPA Axis	**Prolonged critical illness:** - Lower than expected cortisol levels - Loss of pulsatile ACTH release **ME/CFS:** - Lower cortisol baseline - Blunted HPA axis response to stressors - Increased negative feedback - Loss of morning ACTH peak	**Hypothalamus:** cytokine-mediated increase in abundance and affinity of glucocorticoids receptors (GRs) inhibits CRH release **Pituitary:** increase in abundance of GRs inhibits ACTH release **Adrenal gland:** adrenal atrophy (due to lack of pulsatile ACTH stimulation during acute phase)
HPS Axis	**Prolonged critical illness:** - Loss of pulsatile GH release - Low or normal IGF-1 **ME/CFS:** - Low nocturnal GH - Mixed response to stimulation - Failed response to exercise (fibromyalgia) - Low or normal IGF-1	**Hypothalamus:** lack of stimulation by ghrelin; change in relative amounts of hypothalamic stimulating/inhibiting hormones (GHRH/GHIH) dampens GH release **Pituitary**: lack of stimulation by ghrelin dampens GH release
HPT Axis	**Prolonged critical illness** - Loss of pulsatile TSH release - Lower T3 - (Lower T4) - Higher rT3:T3 **ME/CFS:** - (Lower Free T3) - Higher rT3:T3	**Hypothalamus:** cytokine-induced alteration in set-point for release of TRH (local upregulation of T4 to T3 conversion) **Pituitary:** cytokine-mediated suppression of TSH secretion **Thyroid gland:** cytokine-mediated reduction in T4 secretion by the thyroid gland. **Periphery:** upregulation of T3 to rT3 conversion (notably in liver), tissue specific alteration in T3 uptake (i.e., reception and transport), etc.

## A “Vicious Circle” Perpetuating Illness

**Figure 4:**
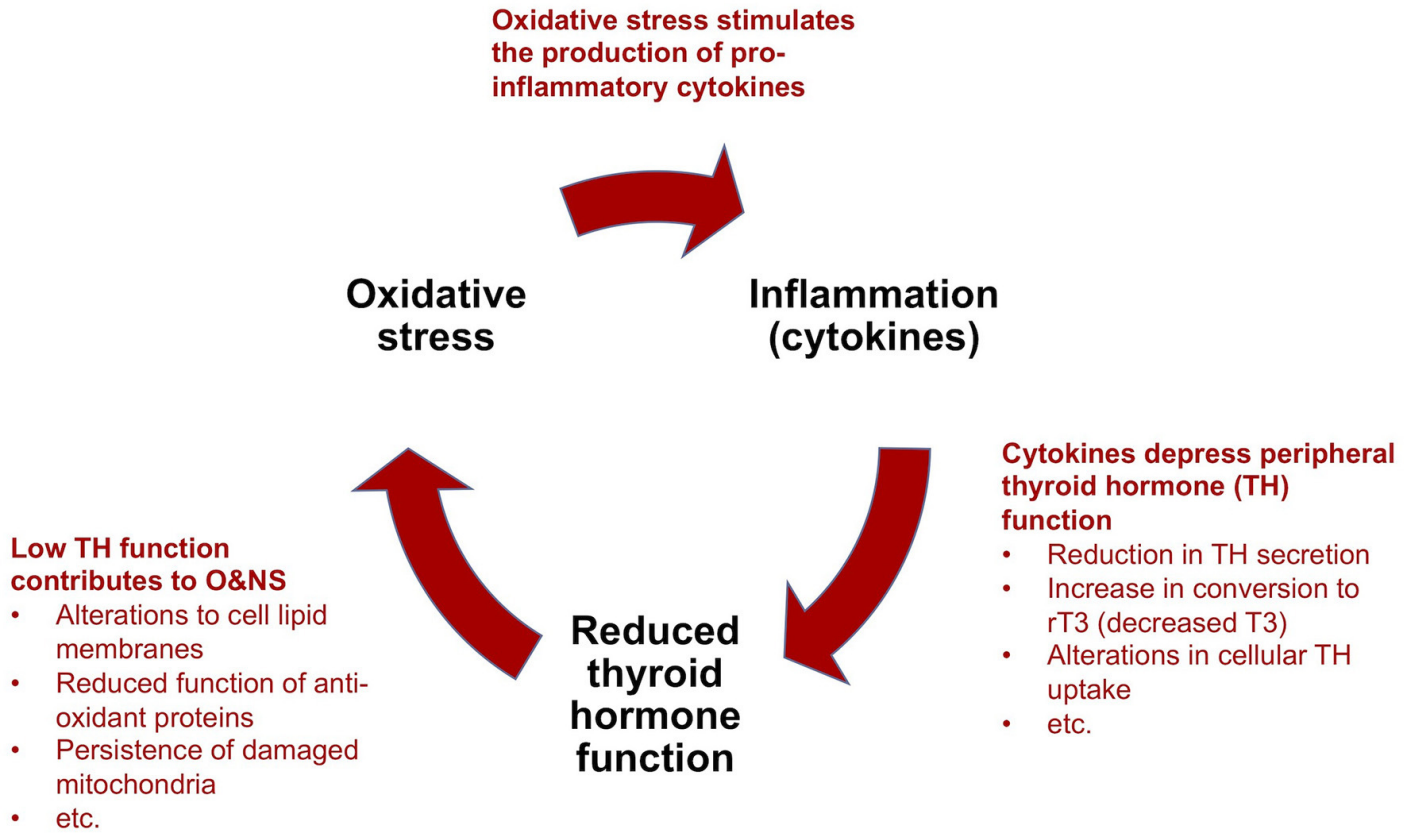
Simplified model to explain the perpetuation of *prolonged* critical illness: a “vicious circle”.

Based on nearly five decades of research, critical illness researchers have proposed a model that describes how NTIS is maintained by reciprocal relationships between inflammation (notably pro-inflammatory cytokines), O&NS and reduced thyroid hormone *function*, forming a “vicious circle” (9, 10) (Figure 4). This model can help to explain the perplexing failure to recover of some critically ill patients in ICUs that survive their initial severe illness or injury. We describe the main elements of this model in a simplified manner below, as well as the implications for metabolism and the immune system. We also provide evidence suggesting that the “vicious circle” observed in *prolonged* critical illness also underlies ME/CFS.

### In Prolonged Critical Illness

The key elements of the suggested “vicious circle” in *prolonged* critical illness include the following mechanisms:

**(a) Cytokines depress thyroid hormone function:** As described above [see section The thyrotropic axis (HPT Axis) In Critical Illness], in *acute* and early stages of critical illness, various peripheral mechanisms involving cytokines lead to the quick depression of thyroid hormone activity in tissue-specific ways. In *prolonged* critical illness, cytokines in association with other signaling factors targeting the hypothalamus, as well as the pituitary and the thyroid glands, also inhibit thyroid hormone production. The relative sequence and importance of these various mechanisms in depressing the HPT axis and thyroid hormone *function* in different tissues and phases of critical illness are the subject of most NTIS publications (10, 104, 105). Notwithstanding the effect of other mechanisms, alterations in the activity of the deiodinase enzymes lead to a decrease in T3 and an increase in rT3 and thus a reduction in thyroid hormone *function* in peripheral tissues during *prolonged* critical illness [based on biopsies on ICU patients who died (142) and studies on mice (143, 144)]. Circulating thyroid hormone concentrations, however, only reveal the “tip of the iceberg” of the alterations occurring at the tissue level (141, 145), which thus are often missed altogether in clinical settings (146).

**(b) Low thyroid hormone function contributes to oxidative and nitrosative stress:** The relationship between thyroid hormone *function* and O&NS is complex, and both hyperthyroidism and hypothyroidism have been associated with oxidative stress (147). Nonetheless, it seems clear that depressed thyroid hormone *function* hinders tissue cells from maintaining a healthy O&NS balance. Mechanisms include alterations to the lipid concentration of the cell membranes that maintain the cell's O&NS balance (148), and reduced function of two proteins (Uncoupling Proteins-2 and -3) with anti-oxidant properties (149). Moreover, in low thyroid hormone *function* conditions, mitochondria damaged by O&NS are not cleared out of cells (9). In turn, it appears that oxidative stress depletes the glutathione required by the abovementioned deiodinase enzymes for the conversion of T4 into T3 (104). Similarly, competition for, and the resulting depletion of the trace mineral selenium—a component of both the deiodinase and the anti-oxidant enzymes (150) —may amplify the self-perpetuating link between increased oxidative stress and low thyroid hormone *function*.

**(c) Oxidative and nitrosative stress stimulate the production of pro-inflammatory cytokines**: The final mechanism which completes the “vicious circle” in *prolonged* critical illness is the link between O&NS and inflammation. O&NS stimulates the production of pro-inflammatory cytokines, notably leptin, resistin, TNF-α and IL-6 (151). In turn, pro-inflammatory cytokines (notably IL-6) further increase O&NS by triggering the production of superoxide radicals (104, 152). There is thus a tendency for O&NS and pro-inflammatory cytokines to perpetuate each other as well.

In sum, according to a model proposed by critical illness researchers, a “vicious circle” involving O&NS, pro-inflammatory cytokines, and low thyroid hormone *function*—as well as reciprocal relationships across these elements—can perpetuate a hypometabolic and inflammatory state, and thus help to explain why some critically ill patients fail to recover.

### In ME/CFS

Similar patterns of O&NS, cytokines, and low thyroid hormone *function* have recently been documented in ME/CFS patients providing the elements for a similar “vicious circle.” We briefly summarize the findings from ME/CFS research relevant to each of these elements.

**Reduced thyroid hormone function:** An immune-mediated loss of thyroid hormone *function* in ME/CFS has long been suspected (132). As mentioned above [see section: The thyrotropic axis (HPT Axis) In ME/CFS], a recent study confirmed that CFS patients have lower circulating levels of Free T3, Total T4, and Total T3 than controls (141). Moreover, this study found a significantly higher ratio of rT3 to T3 hormones. These findings imply a depressed thyroid hormone *function* resembling NTIS. Given the possible tissue-specific alterations in thyroid hormone activity resulting from peripheral mechanisms, the authors suggest these circulating levels only reflect the “tip of the iceberg” of genuine T3 deficits in target tissues.

**Oxidative & nitrosative stress:** Numerous studies have found increased O&NS in ME/CFS and identified this as a factor in the observed metabolic dysfunction (153, 154). Indeed, Pall proposed a model that describes a “vicious circle” involving oxidative stress and cytokines in ME/CFS a decade ago (cf. the “NO/ONOO-Cycle”) (155). Researchers also suggest that high lactate and low glutathione levels found in the brains of ME/CFS patients likely derive from similar mechanisms involving oxidative stress (156). A recent study described the relationship between O&NS and immune-inflammatory pathways in ME/CFS (80).

**Pro-inflammatory cytokines:** Neuro-inflammation is central to ME/CFS, and many researchers have tried to develop diagnostic biomarkers for ME/CFS based on cytokine profiles of patients (157, 158). Montoya et al. found that some 17 cytokines were positively correlated with the severity of ME/CFS, of which 13 are pro-inflammatory. Similarly, circulatory levels of pro-inflammatory cytokines are altered in fibromyalgia patients (159). However, others have argued that given the innumerable sources of potential variance in the measurement of cytokines, it is “unlikely that a consistent and replicable diagnostic cytokine profile will ever be discovered” for ME/CFS (160). It may therefore be ineffectual to compare the cytokine profiles of ME/CFS and *prolonged* critical illness patients.

In sum, given the presence of reduced thyroid hormone *function*, O&NS and pro-inflammatory cytokines in ME/CFS, the “vicious circle” model proposed by critical illness researchers to explain *prolonged* critical illness may also help to understand why ME/CFS patients fail to recover.

### Implications of the “Vicious Circle” and Its Elements

Reduced thyroid hormone *function*, increased O&NS and pro-inflammatory cytokines discovered in *prolonged* critical illness as well as in ME/CFS have important implications notably on metabolism, organ function, immune responses and the endocrine system. These are further described below:

**Reduced thyroid hormone function:** The *prolonged* down-regulation of thyroid hormone activity certainly has implications for the immune system. Authors describe the profound effects of circulating thyroid hormone levels on the activity of monocytes, lymphocytes macrophages, neutrophils, dendritic cells and natural killer cells; as well as cytokines (161–170). Notably, depressed thyroid levels appear to depress the activity of natural killer cells (171)—a signature finding in ME/CFS (172). Such immune dysfunctions might explain other pathologies, such as viral reactivation observed in ICU patients (173–175) and suspected in ME/CFS patients (176, 177). Experimenting on rats, researchers have shown that depressed thyroid hormone levels occur in a specific sequence, manifesting (from first to last) in the liver, kidney, brain, heart and adipose tissues (145). An implication of a tissue-specific down-regulation of thyroid hormone activity is differential impact on organ function. Some ME/CFS practitioners have argued that tissue-specific modulation of T3 can help explain the disparate and evolving symptoms in ME/CFS and fibromyalgia (133, 134, 138, 140). In aggregate, depressed thyroid hormone *function* would engender a general hypometabolic state. Finally, thyroid hormone *function* also impacts other endocrine axes as well (178, 179)—notably the HPA axis—setting the stage for further complex interactions between the various endocrine axes and the immune system.

**Oxidative & nitrosative stress:** The implications of chronic oxidative stress in the body are widely documented. In addition to inducing inflammation, oxidative stress causes cell damage and disrupts normal cellular transcription and signaling mechanisms (9). O&NS has been shown to cause mitochondrial damage during critical illness (180) and ME/CFS (153).

**Pro-inflammatory cytokines:** Researchers are finding that the more than 100 different cytokines play a part in determining the function of hormones through both central and peripheral mechanisms (32). As described in the previous section, cytokines are likely culprits in the central (i.e., hypothalamic and pituitary) suppression of the HPA, HPS and HPT axes in *prolonged* critical illness (29). Pro-inflammatory cytokines and inflammation also hinder normal mitochondrial function during critical illness (181). The alterations in cytokines found in critical illness likely have many further implications that have yet to be fully understood (182) which is also the case for ME/CFS (183).

### Intermediate Conclusions

In sum, critical illness researchers have proposed that the self-perpetuating relationships between inflammation (notably pro-inflammatory cytokines), O&NS and low thyroid hormone *function* explains the maintenance of illness in some ICU patients following severe injury or infection. Given that the same elements of such a “vicious circle” have also been documented in ME/CFS, we suggest that the model can also explain the failure of ME/CFS patients to recover. Moreover, these elements have been shown to have profound implications on metabolism, as well as on the function of the immune and endocrine systems—which in in turn could explain the myriad of symptoms in *prolonged* critical illness and ME/CFS.

## Relationship to Other Hypotheses of ME/CFS Pathogenesis

Our hypothesis that maladaptive mechanisms which prevent recovery in *prolonged* critical illness also underlie ME/CFS complements several other hypotheses of ME/CFS pathogenesis. In this section we provide an initial and non-exhaustive discussion of some of these complementarities.

**Allostatic overload:** Some researchers consider ME/CFS to be a maladaptive response to physical, infectious, and/or emotional stressors. They describe an “allostatic overload” (i.e., the cumulative effect of stressful situations exceeding a person's ability to cope) or a “‘crash’ in the stress system” (184, 185). Our hypothesis fits into this theoretical framework and offers an explanation for the possible underlying physiological mechanisms by drawing on the research from critical care medicine.

**Hypothalamic endocrine suppression:** Researchers have suggested that hypothalamic endocrine suppression could explain ME/CFS (132, 186) and fibromyalgia (187–189). Our thesis upholds this hypothesis and seeks to strengthen it by suggesting that the controversy around the existence of central endocrine suppression in ME/CFS may be resolved by studying the *pulsatile* secretions of the pituitary—rather than single or average measurements of circulating tropic and non-tropic hormone concentrations, which can fail to discern the dysfunctions of the endocrine axes.

**Anomalies in thyroid hormone function:** Numerous clinical practitioners and researchers believe that anomalies in thyroid hormone *function*—including changes in the conversion of thyroid hormones, a resistance of thyroid hormone receptors at cellular level, etc. —contribute to ME/CFS and fibromyalgia (133–141). Indeed, practitioners have written about their successes in treating ME/CFS patients with thyroid hormone supplements (42, 77, 188, 190–194); and patients have published books on their experiences (195–197). Our hypothesis complements this reasoning: we propose that both the central and peripheral mechanisms altering thyroid hormone *function* during critical illness (c.f. NTIS, euthyroid sick syndrome or “low T3 syndrome”) also occur in ME/CFS. Moreover, by applying a model from critical illness, we suggest that low thyroid hormone *function* is one element of a “vicious circle” perpetuating illness in ME/CFS.

**Viral Reactivation:** It has long been suggested that viral reactivation plays a role in ME/CFS, particularly reactivation of Epstein-Barr virus (EBV) and cytomegalovirus (CMV) (176, 177). Similarly, high incidences of viral reactivation have also been observed in ICU patients, notably in patients with sepsis and *prolonged* critical illness. ICU researchers propose that this viral reactivation is a result of immune suppression occurring during critical illness (173–175). Thus, critical illness research would suggest that viral reactivation is a secondary pathology in ME/CFS—except in cases in which the viral infection was the onset event.

**Viral infection:** Viral infection is recognized to be a leading onset event of ME/CFS (16, 198–201). This is particularly concerning in the context of the COVID-19 pandemic. Many COVID-19 patients continue to experience a variety of debilitating symptoms after defeating the virus that resemble ME/CFS. Building on our hypothesis, we would suggest that post COVID-19 syndrome is evidence of a maladaptive response to the stress of infection akin to that experienced in *prolonged* critical illness and ME/CFS.

**Chronic inflammation:** Researchers have found that chronic inflammation—auto-immune, allergic or bacterial/viral—underlies ME/CFS (194, 202, 203). Others also ascribe the perpetuation of ME/CFS to the relationship between inflammation and O&NS (80, 155). Our hypothesis is largely complementary to these findings and associated theories. Indeed, following a cytokine surge during the *acute* phase of critical illness, inflammation is believed to persist in the case of *prolonged* critical illness (4). Moreover, pro-inflammatory cytokines and O&NS are elements in the “vicious circle” model of *prolonged* critical illness, which we propose also serves to understand the perpetuation of illness in ME/CFS patients.

**Neuroinflammation of the brain:** ME/CFS is associated with inflammation of the brain (hence the name myalgic encephalomyelitis) (204, 205). Some have specifically proposed that inflammation of the hypothalamus underlies ME/CFS (81, 82). Similarly, alterations of the endocrine axes through mechanisms mediated by pro-inflammatory cytokines which impact the hypothalamus and pituitary are central to *prolonged* critical illness (see section Suppression of Pulsatile Pituitary Secretions).

**Energy metabolic defect:** Researchers have found impairment in energy production (205, 206), reduced mitochondrial activity (207–209) and irregularities in the metabolites of ME/CFS patients (210, 211) —suggesting that they experience a hypometabolic or “dauer” state (212). Our hypothesis is compatible with analyses that emphasize metabolic defects in ME/CFS. Indeed, the suppression of pituitary secretions, depressed thyroid hormone *function*, O&NS and immune system dysfunction—hallmarks of *prolonged* critical illness—have severe impacts on metabolism, including on glucose utilization and mitochondrial activity (see section A “Vicious Circle” Perpetuating Illness). Certainly, *prolonged* critical illness resembles a hypometabolic “dauer” state as well.

**Genetic predisposition:** Research also suggests there may a genetic element in the pathogenesis of ME/CFS (213–216). Our hypothesis is compatible with a possible genetic predisposition for ME/CFS. Indeed, it is not known why some critically ill patients succumb to *prolonged* critical illness while others begin recovery (217, 218); genetics may play a role. The findings from the field of ME/CFS in the area of genetics might inform the field of critical illness in this regard.

In sum, our hypothesis is largely complementary to hypotheses that emphasize metabolic, hormonal and/or immune dysfunctions in the pathogenesis of ME/CFS. Our hypothesis—drawing from research on critical illness—integrates these dysfunctions into a single framework and provides arguments for the direction of causality between them.

## Conclusion

Decades of research in the field of critical medicine have demonstrated that in response to the stress of severe infection or injury, endocrine axes experience profound alterations. An assessment of the pituitary's *pulsatile* secretions reveals that in the subset of patients which survive their severe infection or injury but do not begin recovery (i.e., *prolonged* critically ill patients), the suppression of endocrine axes is maintained irrespective of the initial severe infection or injury. Recent pathological models propose that mechanisms involving pro-inflammatory cytokines, O&NS and low thyroid hormone *function* explain the perpetuation of these endocrine dysfunctions (i.e., a “vicious circle”).

The symptoms, physiological abnormalities and endocrine patterns observed in severe ME/CFS are not unlike those of *prolonged* critical illness. Moreover, the same elements of a “vicious circle” also exist in ME/CFS. However, unlike in critical illness, the pituitary's *pulsatile* secretion and its relationships to metabolic and immune functions remain largely unstudied in ME/CFS.

Without excluding possible predisposing genetic or environmental factors, we propose the hypothesis that the maladaptive mechanisms that prevent recovery of *prolonged* critically ill patients also underlie ME/CFS. The severity of ME/CFS illness may be a function of the strength of these mechanisms; very severe ME/CFS most resembles *prolonged* critical illness. We further argue that this hypothesis should be investigated through collaborative research projects building on the findings from critical illness and ME/CFS. If this hypothesis is validated, past trials to break the “vicious circle” that perpetuates critical illness, and the early successes to reactivate the *pulsatile* secretion of the pituitary in ICU patients, may provide avenues for a cure for ME/CFS—including cases onset by infections. Certainly, given the similarities described above, active collaboration between critical illness and ME/CFS researchers could lead to improved outcomes for both conditions.

Finally, we suggest that immediate collaborative efforts should be sought among the researcher community in order to conduct longitudinal studies with the aim of identifying similarities and differences across *prolonged* critical illness, post-ICU syndrome, ME/CFS, fibromyalgia and long-COVID in relation to the hormonal axes, O&NS and pro-inflammatory response with the objective of discovering diagnostic and therapeutic targets mitigating the functional disability that these conditions induce.

## Data Availability Statement

The original contributions generated for the study are included in the article/supplementary material, further inquiries can be directed to the corresponding author/s.

## Author Contributions

DS wrote the first draft of the manuscript. All authors contributed to manuscript revision, read, and approved the submitted version.

## Conflict of Interest

The authors declare that the research was conducted in the absence of any commercial or financial relationships that could be construed as a potential conflict of interest.

## Abbreviations

ACTH: Adrenocorticotropic hormone
ARV: Arginine vasopressin
CRH: Corticotrophin-releasing hormone
DHEA: Dehydroepiandrosterone
GH: Growth hormone
GHIH: Growth hormone inhibiting hormone
GHRH: Growth hormone releasing hormone
HPA: hypothalamus-pituitary-adrenal axis: “Adreno-cortical axis”
HPS: Hypothalamic-pituitary-somatotropic axis: “Somatropic axis”
HPT: Hypothalamic-pituitary-thyroid: “Thyrotropic axis”
ICU: Intensive Care Unit
IGF-1: Insulin like growth hormone-1
IGFBP: Insulin like growth hormone binding proteins
ME/CFS: Myalgic Encephalomyelitis/Chronic Fatigue Syndrome
NTIS: Non-thyroidal illness syndrome
O&NS: oxidative and nitrosative stress
PICS: Post-intensive care syndrome
POTS: Postural Orthostatic Tachycardia Syndrome
TRH: Thyrotropin-releasing hormone
TSH: Thyroid stimulating hormone.
